# Retracted: Adrenal Schwannomas: Rare Tumor of the Retroperitoneum

**DOI:** 10.1155/2016/8739489

**Published:** 2016-01-20

**Authors:** Case Reports in Surgery

The paper titled “Adrenal Schwannomas: Rare Tumor of the Retroperitoneum” [1] has been retracted as it is found to contain a substantial amount of material, without referencing, from the article titled “Laparoscopic Resection of an Adrenal Schwannoma” by Toutouzas G. Konstantinos, Tsamis Dimitrios, Kekis B. Panagiotis, Michalopoulos V. Nikolaos, Flessas Ioannis, Manouras Andreas, and Zografos Geogrios, published in the Journal of the Society of Laparoendoscopic Surgeons.
